# Awareness of age-related gains and losses as moderators of daily stress reactivity in middle- and older-adulthood

**DOI:** 10.3389/fpsyt.2022.929657

**Published:** 2022-08-26

**Authors:** Bethany Wilton-Harding, Nathan Weber, Tim D. Windsor

**Affiliations:** ^1^College of Nursing and Health Sciences, Flinders University, Adelaide, SA, Australia; ^2^College of Education, Psychology and Social Work, Flinders University, Adelaide, SA, Australia

**Keywords:** affect, wellbeing, awareness of aging, daily stressors, subjective aging, daily diary

## Abstract

**Objectives:**

Associations between awareness of one’s own aging and wellbeing have received increasing attention in the field of gerontology over the last decade. The current study examines how between-person differences and within-person fluctuations of awareness of age-related change (AARC) relate to daily negative affect and vitality. Of key interest was the extent to which fluctuations in AARC moderated reactivity to stressor exposure. We predicted that higher positive perceptions of aging (AARC-gains) would buffer the relationship between daily stressors and negative affect/vitality. Conversely, we expected that higher negative perceptions (AARC-losses) may exacerbate the relationship between daily stressors and the outcome variables.

**Methods:**

Data were collected from a community-based sample of 152 Australian adults aged 53–86 (*M* = 69.18, *SD* = 5.73). For 10 consecutive days, participants completed surveys on their smartphones measuring daily stressors, AARC, and affect (positive and negative). Bayesian hierarchical linear models were used to examine whether AARC-gains and AARC-losses moderated within-person associations of daily stressors and affect (i.e., stress reactivity).

**Results:**

At the between-person level, higher AARC-gains was associated with lower negative affect and higher vitality, whereas those reporting higher AARC-losses scored higher on negative affect and lower on vitality. Within-person variables revealed that on days when AARC-gains was higher and AARC-losses was lower, this corresponded with lower negative affect and higher vitality. There was no evidence in support of individual moderating effects of within-person AARC-losses or within-person AARC-gains on stress reactivity. A trend was evident in support of a three-way WP Stress severity × WP AARC-gains × WP AARC-losses interaction in the prediction of negative affect, indicating that on days when AARC-losses was higher, the association of stress severity with negative affect was weaker if AARC-gains was higher. Follow-up analyses modeling quadratic stress severity revealed a trend suggesting an interaction of within-person stress severity and within-person AARC-losses.

**Discussion:**

Results indicate that both individual differences and short-term fluctuations in AARC are associated with daily negative affect and vitality. The results provided qualified support for a possible protective role of AARC-gains in the context of stress reactivity.

## Introduction

Across the lifespan, individuals hold beliefs about older people, old age itself, and their own experiences of aging. Such beliefs- referred to as views on aging- are shaped by biological-evolutionary, psychological, and social-contextual influences, and are regarded as both drivers and outcomes of lifespan development (1). The significance of views on aging for aspects of health and psychosocial functioning is supported by numerous empirical studies. For example, both a greater awareness of age-related gains, and less awareness of age-related losses have been linked with higher wellbeing [e.g., (2–4)]. Recently, studies have further contributed to our understanding of dynamic processes linking short-term changes in subjective aging with wellbeing outcomes using micro-longitudinal study designs (5, 6). In the current study, we add to this emerging area of research by using daily diary data from a sample of midlife and older Australians to examine within-person processes linking stress exposure to daily fluctuations in affect and vitality. Of central interest was whether participants appeared less reactive to stress (with reactivity indicated by higher negative affect and lower vitality) on days when they reported relatively higher awareness of age-related gains, and relatively lower awareness of age-related losses.

### Reactivity to daily stressors in older adulthood

Daily stressors refer to everyday hassles that can occur in day-to-day life. While such experiences are unlikely to impact wellbeing to the same extent that major life events can, everyday stressors may nevertheless have a deleterious cumulative effect over time (7). On days when individuals report experiencing a stressor, they typically exhibit a corresponding increase in negative affect [and/or decrease in positive affect (8, 9)]. How individuals respond to daily stressors appears to have long-term implications for mental health, with research indicating that greater affective reactivity to stress increases the likelihood of reporting an affective disorder a decade later (10).

Research evidence regarding age differences in reactivity to stressors is mixed (11). Some studies point to older adults having fewer stressors, and perceiving stressors as less severe than younger and middle-aged adults (12–14), possibly as a result of older adults’ proficiency in emotion regulation (15, 16). Conversely, other studies have shown higher affective (17), and physical (18) reactivity to daily stress among older relative to younger adults. Older adulthood is a time of substantial developmental heterogeneity (19), and growing older is experienced differently both between individuals (20), and within individuals over time (5). The current research moves beyond a focus on chronological age differences in stress reactivity, by considering whether stress reactivity may vary as a function of daily perceptions of age-related change.

### Awareness of age-related change

Relatively recently in the subjective aging literature, Diehl and Wahl (21) conceptualized the model of awareness of age-related change (AARC). AARC refers to “all those experiences that make a person aware that his or her behavior, level of performance, or ways of experiencing his or her life have changed as a consequence of having grown older” [(21) p. 340]. AARC is one of the first multidimensional models of subjective aging to distinguish between perceptions of both positive (AARC-gains) and negative (AARC-losses) aspects of growing older.

While AARC has been predominantly considered a more trait-like construct in previous literature, it has recently been found that perceptions of both age-related gains and losses fluctuate across short time scales [i.e., daily over 8 consecutive days, (5, 6)]. Furthermore, negative affect was found to increase on days when AARC-losses was higher-than-usual (5). In another study, the relationship between stressors and negative affect was found to be moderated by general attitudes toward one’s own aging assessed at the between-person (trait) level. Here, reactivity to daily stressors was stronger among those who had generally more negative attitudes toward their own aging (22). In the current study, we aim to further extend this emerging area of work by considering daily AARC-gains, AARC-losses and their interaction as possible moderators of affective reactivity to daily stressors.

### Awareness of age-related change and reactivity to daily stressors

There are several theoretical arguments as to why daily fluctuations in AARC may have implications for stress reactivity. First, Diehl and Wahl (21) proposed that AARC is linked to other self-relevant constructs which are implicated in the stress reactivity process, such as perceived control, self-efficacy, self-concept clarity, and self-representation. Second, societal stereotypes regarding aging are often internalized by older adults (23), which can lead to negative self-stereotyping, which may have implications for behavioral outcomes (21). Subliminally primed age-related self-stereotypes have been shown to impact walking speed (24), memory (25), handwriting (26), and the endorsement of hypothetical life-prolonging interventions (27). Furthermore, individuals exposed to negative aging stereotypes showed greater physiological response to stress compared to those exposed to positive stereotypes (28). It follows that on occasions when people perceive their own aging negatively, they may be less likely to engage in active coping or to engage with new goals [e.g., (29)] in response to potential stressors in ways that support wellbeing. Conversely, positive perceptions of aging may enable an appreciation of current experiences and resources (30), and better support processes of self-regulation that facilitate coping (21, 29, 31, 32).

In terms of self-relevant psychosocial resources, perceived control has received some research attention as a moderator of affective reactivity to stressors at the daily level. Overall, reactivity to stressors appears to be lower on days when individuals perceive higher-than-usual personal control (33, 34). As awareness of aging impacts individuals’ social contexts, expectations, behavior, and resources (35), AARC is likely to also influence perceptions of personal control (36). AARC and control beliefs have been found to covary at the daily level, such that on days when AARC-gains was higher-than-usual, and on days when AARC-losses was lower-than-usual, individuals reported higher control beliefs (36).

Given that more positive perceptions of aging have been linked to adaptive outcomes, higher-than-usual AARC-gains could act as a resource that protects individuals from reactivity to stressors (22, 28). Higher-than-usual AARC-gains may enable individuals to evaluate their aging in a more favorable light (21), affording continued opportunities for growth which could provide respite from negative events (30). While AARC-gains has been shown to vary significantly within-person (5), a novel aspect of the current research is that we examine this further by considering how AARC-gains relates to daily wellbeing, and the extent to which it buffers against stress reactivity. Specifically, we expected that reactivity to daily stressors (evidenced by higher negative affect and lower vitality) would be weaker on days when participants report higher-than-typical AARC-gains.

Conversely, daily experiences which bring awareness to possible future limitations due to aging (i.e., AARC-losses) may prompt individuals to feel less in control of their life and outcomes (2, 32, 37). Furthermore, higher-than-usual AARC-losses may lead to identification with internalized negative aging stereotypes (38), which is linked to increased stress reactivity (28). Hence, we expected reactivity to daily stressors would be greater on days when participants report higher-than-typical AARC-losses.

### The present study

In the present study we considered daily stressor severity and between- and within-person differences in AARC as predictors of daily negative affect and vitality. Negative affect is commonly used as an index of reactivity to stressors in daily diary research [e.g., (22)]. We also considered vitality as an additional marker of psychological wellbeing likely to fluctuate on a day-to-day basis, that could be undermined by exposure to stressors. Subjective vitality refers to the feeling of being alive or energized (39), and recent conceptualizations on lifespan development have emphasized the role of subjective energy as an important driver of goal pursuit (40), health behaviors (23) and social engagement (41).

At the between-person level, we expected that participants reporting higher overall levels of AARC-gains, and lower overall levels of AARC-losses would also report higher overall vitality and lower negative affect. Of greater specific interest in the current study were within-person associations: here we expected that the relationship of daily stressor severity with vitality and negative affect would vary as a function of both daily AARC-gains and AARC-losses. Specifically, we expected the predicted associations of stressor severity with higher negative affect and lower vitality to be weaker on days when AARC-gains was higher than usual, and to be stronger on days when AARC-losses was higher than usual.

Finally, recent cross-sectional studies conducted by our group using different data sources have shown that- at the between-person level- negative associations of AARC-losses with wellbeing tend to be weaker among those who perceive relatively higher AARC-gains, suggesting that perceived gains might play a role in off-setting detrimental effects of perceived losses on wellbeing (42). We also examined interactions of daily AARC-gains with AARC-losses in the present study, to consider a possible buffering effect of AARC-gains. Specifically, we predicted that the association of daily AARC-losses with stress reactivity would be weaker on days when AARC-gains was relatively higher, compared with days when AARC-gains was relatively lower.

## Materials and methods

### Participants and procedure

A community-based sample of 163 middle-aged and older adults was recruited through distribution of a study advertisement in May-June 2020 to our lab’s participant database, and to online networks of older Australian adults. Participants aged over 50, who had regular access to a smartphone with text message/internet capability were eligible to participate. After completion of an online baseline survey, participants were given a unique, randomly generated code, then redirected to sign up to the registration page for the mobile component of the study, a short message service (SMS) platform to receive daily Qualtrics survey links *via* text message [SurveySignal; (43)]. Of the 163 eligible individuals who registered interest in participating, five did not proceed to the baseline survey. Data from one individual who self-reported a diagnosis of mild cognitive impairment/dementia were excluded. A further five individuals did not complete any evening surveys. The final sample consisted of 152 participants aged between 53 and 86 (*M* = 69.18, *SD* = 5.73). Approximately two-thirds of participants were female (66.4% female; 33.6% male; 0% non-binary), and the majority were retired (85.5%). Around half (49.3%) of participants reported completion of tertiary education. Participants were approximately 98.7% Caucasian, and 1.3% Asian Australian. Over two-thirds of respondents were partnered (67.8%). The study was approved by the Flinders University Human Research Ethics Committee (Project #8368).

For 10 consecutive days, participants received a link to the evening survey at approximately 8:00 p.m., which was available for 3 h. The survey measured daily stressors, AARC, vitality and affect. Participants also received survey links at randomly spaced intervals four times per day as part of a larger study [see (44)], however, the variables reported here were only assessed in the evening surveys. A response-based compensation approach was offered as follows: Participants received $10 AUD as base compensation, with tiered incentives offered for greater completion of daily surveys (over 60% = an additional $25, over 80% = an additional $40). Total possible compensation was $50 AUD per participant. For the 10 evening measurements, participants completed an average of 8.33 surveys (range 1–10), with over 80% of participants completing eight or more assessments, totaling 1,266 observations.

### Measures

#### Awareness of age-related change

Daily AARC was measured using the AARC-10 SF (45), with two five-item subscales each measuring AARC-gains and AARC-losses across the domains of health and physical functioning, cognitive functioning, interpersonal relations, social-cognitive and socio-emotional functioning, and lifestyle and engagement (21). Following Neupert and Bellingtier (5), participants were presented with the item stem “with my awareness of aging today…” and asked to rate on a scale from 1 (*not at all*) to 5 (*very much*), the extent to which each item applied to them that day (e.g., “…I have a better sense of what is important to me”). Higher scores represent greater daily AARC-gains (day 1 Cronbach’s α = 0.68, day 10 α = 0.78) and AARC-losses (day 1 α = 0.76, day 10 α = 0.87). To determine the degree of day-to-day variation in AARC, we fitted variance components models (i.e., multilevel models with no predictor variables) for AARC-gains and AARC-losses. These analyses indicated that 25 and 23% of the variance occurred at the within-person level for AARC-gains and AARC-losses, respectively.

#### Daily stressor severity

Daily stressors were assessed with a modified version of the Daily Inventory of Stressful Events (46, 47). Participants were asked to report whether they had experienced any of five stressors in the previous 24 h: (1) an argument or disagreement; (2) potential argument or disagreement that was allowed to pass; (3) an event affecting a friend or relative that was stressful; (4) health-related stressor; (5) another stressor. Where participants endorsed a stressor, they also provided a rating of its severity on a 5-point scale (1 = *not at all stressful*, 5 = *very stressful*). For the current analysis, we calculated a general index of daily stress severity by summing the severity ratings for each day (stressors not endorsed were coded as “0”). Across the sample, the number of stressful events reported was relatively low (Means across days 1–10 ranged from 0.46 to 0.72 stressful events).

#### Negative affect

Daily affect was measured using the Scale of Positive and Negative Experiences [SPANE; (48)]. The 12-item scale required participants to rate the extent to which they experienced both positive and negative feelings (e.g., “happy,” “contented,” “angry,” “unpleasant”) in the previous 24 h on a scale from 1 (*very rarely or never*) to 5 (*very often or always*). Consistent with previous research on daily stress reactivity [e.g., (22)] we focus on negative affect in the current analysis. Higher scores reflect higher daily negative affect (day 1 α = 0.85, day 10 α = 0.88).

#### Vitality

Vitality was measured using the Subjective Vitality Scales (39, 49) with items slightly modified for administration using a daily diary format. Participants indicated their agreement with six statements regarding their subjective energy levels in the past 24 h (e.g., “I felt alert and awake”) on a scale from 1 (*not at all true*) to 7 (*very true*). Higher scores represent higher subjective vitality (day 1 α = 0.93, day 10 α = 0.95).

#### Covariates

Analyses were adjusted for time-related effects, including day in study (coded as 0–9), and weekend (0 = weekday, 1 = weekend). We also controlled for baseline chronological age, gender (0 = male, 1 = female), education (0 = completed tertiary education, 1 = did not complete tertiary education) and labor force status (0 = in the labor force, 1 = not in the labor force). Physical functioning was measured using the 10-item physical functioning subscale from the RAND health survey (50). Participants rated the extent to which their health limits them from participating in certain activities (e.g., climbing several flights of stairs) on a three-point scale with answers ranging from “no, not limited at all” to “yes, limited a lot”; recoded scores range = 0–100, *M* = 79.77, *SD* = 20.46, α = 0.90; with higher scores reflecting better physical functioning. These variables were statistically controlled due to previous associations with subjective aging (20), stressor experience (10) and affective wellbeing (15).

### Statistical analysis

Given the hierarchies inherent in daily diary studies (i.e., measurement occasions at Level 1 nested within participants at Level 2), data of this type are typically analyzed using hierarchical models that simultaneously model variance at each of the between-person (Level 2–BP) and within-person (Level 1–WP) levels [e.g., (51)]. We applied this approach in the present study, fitting two-level models with a random intercept, but departed from more commonly used frequentist methods by generating Bayesian parameter estimates. Models were fit using Stan (52) *via* the R (53) package brms (54, 55). Additionally, data and results processing were conducted using the tidyverse (56), tidybayes (57), and cowplot (58) packages. Bayesian inference offers several advantages over traditional null hypothesis testing, including the ability to directly assess the credibility of both the null and alternative hypotheses, and the scope to make more nuanced judgments about the reliability of the effects of interest than is possible from a reliance on *p*-values with an arbitrary cut-off (59, 60). All models were fit with continuous variables (outcome and predictor) standardized (*M* = 0, *SD* = 1).

We modeled vitality as a left and right censored (at 1 and 5, respectively, on the original scale) normal distribution. The location (mean) and scale (standard deviation) were estimated with an identity link function. We used weakly informative priors. Specifically, normal (*M* = 0, *SD* = 1) priors were used for the intercept and coefficient for each predictor, and half-t (location = 0, scale = 2.5, df = 3) for the residual standard deviation and as the hyper-prior for the standard deviation of the “random effect” (intercept varying by person-specific ID). Negative affect was modeled using a left censored (at 1 on the original scale) skewed normal distribution. Model specifications were the same as those described above for vitality with the addition of the skewness parameter being estimated *via* an identity link with a normal (*M* = 0, *SD* = 4) prior.

We report regression coefficients (slopes) as the parameters of key substantive interest, with associated Bayesian highest density intervals (HDI_95%_). The width of HDI_95%_ represents the range of most credible values that encompass 95% of the distribution of possible parameters (61). To determine whether the evidence in our data favored the null or alternative hypotheses, we considered the HDI_95%_ in combination with the region of practical equivalence, or ROPE (61). The ROPE represents a range of values that are regarded as being practically equivalent to the null hypotheses (in the case of regression slopes, equivalent to zero). All continuous data were standardized prior to analyses based on the sample *M* and *SD* values with the data in long-form, and binary predictors were coded (−1, 1). We set a ROPE of ± 0.05 for analysis of main effects consistent with recommendations for correlational analysis (61, 62). Categorical inferences are made by comparing the HDI_95%_ and the ROPE. Support, at the 95% level, for the alternative (null) hypothesis is provided when the HDI_95%_ falls entirely outside (inside) the ROPE. Acknowledging the power limitations inherent in testing interaction terms (63) when assessing the reliability of cross-products we compared HDI_95%_ values against both the standard ROPE (± 0.05) and a less conservative ROPE of ± 0.025. Further, to provide a more nuanced index of support for the hypothesis (null), we calculated the proportion of credible parameter estimates that lay outside the ROPE in the observed direction (inside the ROPE). These proportions reflect the probability of a meaningful (null) effect and are denoted as P_meaningful_ (P_null_).

To test our hypotheses, predictor variables of substantive interest (stress severity, AARC-gains, AARC-losses) were disaggregated into BP and WP components. BP variables represented each person’s average across all available assessments (person-mean) and WP variables represented occasion (day) specific deviations from the person mean (64).

## Results

Descriptive statistics and bivariate correlations of associations of the key variables (with data in long-form) are provided in Table 1. The findings of primary interest in relation to negative affect and vitality are discussed in the sections that follow.

**TABLE 1:** Descriptive statistics (at Day 1) and correlations of AARC, stressors, affect, and control variables (data in long-form).

Variable	*M* (*SD*) or %	Range	1	2	3	4	5	6	7	8	9
1. Age	69.18 (5.74)	53–86	–								
2. Gender (female)	66.4%		−0.14	–							
3. Education (University)	49.3%		0.04	0.03	–						
4. Not in the labor force	85.5%		0.34	−0.06	0.11						
5. Physical functioning	79.77 (20.53)	0–100	−0.27	0.00	0.15	−0.11	–				
6. AARC-gains	20.95 (2.62)	12–25	0.08	0.16	0.07	−0.10	0.04	–			
7. AARC-losses	10.13 (3.23)	5–20	0.24	−0.17	−0.05	0.14	−0.53	−0.25	–		
8. Negative affect	2.50 (1.28)	1–3.83	−0.07	0.02	−0.06	−0.09	−0.14	−0.21	−0.35	–	
9. Vitality	4.66 (1.36)	1–7	−0.03	−0.12	0.02	−0.01	0.29	0.32	−0.47	−0.50	–
10. Stress severity	1.63 (3.18)	0–19	−0.08	0.10	−0.04	−0.24	−0.17	−0.11	0.11	0.41	−0.28

N = 152, M, Mean; SD, Standard Deviation; AARC, Awareness of Age-Related Change.

### Negative affect

**FIGURE 1:**
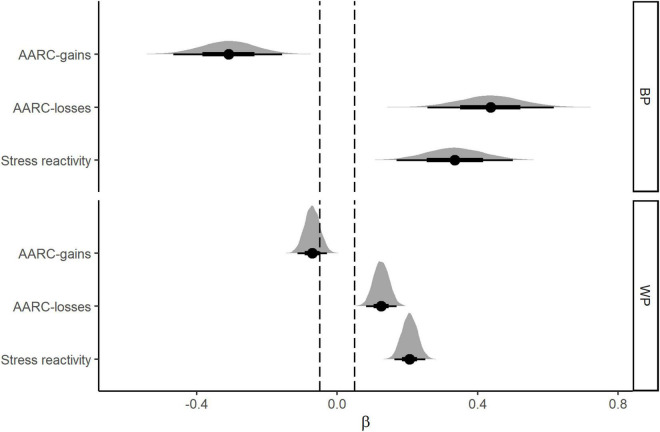
Between-person (BP, **top panel**) and Within-Person (WP, **bottom panel**) main effects for AARC and stress severity as predictors of negative affect. The figure shows posterior distributions of coefficient estimates for the main effects and their degree of overlap with the region of practical equivalence (ROPE_0.05_).

Results from the Bayesian mixed-effects models used to examine associations of daily stress and AARC with negative affect are shown in Table 2. Focusing first on the main effects, consistent with our predictions, there was clear evidence for reliable associations of BP AARC-gains, BP AARC-losses and BP stress with negative affect. In each case the distribution of credible parameter estimates falls entirely outside the ROPE and P_meaningful_ = 1.0. For BP AARC-losses, the coefficient was positive, indicating that participants who on average reported higher AARC-losses across the daily assessments on average reported higher negative affect. The regression coefficient was negative for BP AARC-gains, thus participants who reported greater daily AARC-gains on average, reported lower negative affect. Finally, the positive coefficient for stress severity indicated that those reporting greater stress severity on average reported higher negative affect. The strength and direction of the effects is summarized in “slope-on-a-ROPE” format in Figure 1.

**TABLE 2:** Results of Bayesian hierarchical mixed model predicting negative affect.

	HDI_95%_				ROPE_0.05_			ROPE_0.025_		
Predictor	Est.	Error	Low	High	Below	Within	Above	Below	Within	Above
Intercept	−0.22	0.12	−0.45	0.01						
Age	−0.05	0.08	−0.21	0.11						
Female	0.10	0.08	−0.06	0.26						
Tertiary educated	0.00	0.07	−0.15	0.14						
Not in the labor force	−0.16	0.12	−0.39	0.07						
Physical functioning	0.15	0.09	−0.03	0.32						
Day in study	−0.39	0.10	−0.58	−0.20						
Day in study^2^	0.30	0.10	0.11	0.49						
Weekend	−0.03	0.02	−0.08	0.02						
BP AARC-gains	−0.31	0.08	−0.47	−0.16	1.00	0.00	0.00			
BP AARC-losses	0.44	0.09	0.26	0.61	0.00	0.00	1.00			
BP stress severity	0.33	0.08	0.17	0.49	0.00	0.00	1.00			
WP AARC-gains	−0.07	0.02	−0.11	−0.03	0.84	0.16	0.00			
WP AARC-losses	0.13	0.02	0.08	0.17	0.00	0.00	1.00			
WP stress severity	0.20	0.02	0.16	0.24	0.00	0.00	1.00			
WP AARC gains × WP AARC losses	0.02	0.02	−0.02	0.06						
WP AARC-gains × WP stress severity	−0.02	0.02	−0.05	0.02	0.04	0.97	0.00	0.29	0.69	0.02
WP AARC-losses × WP stress severity	0.00	0.02	−0.04	0.04	0.00	0.99	0.00	0.10	0.82	0.08
WP gains × WP losses × WP stress severity	−0.03	0.01	−0.05	0.00	0.04	0.96	0.00	0.81	0.19	0.00
Random intercept (*SD*)	0.68	0.02	0.65	0.72						

HDI, Highest Density Interval; Est, Estimate; SD, Standard Deviation; BP, Between-person; WP, Within-Person. Predictor and outcome variables were standardized prior to analysis.

Regarding WP effects, there was again strong evidence for non-zero associations of WP AARC-losses and WP stress with daily negative affect, although the effect sizes were smaller relative to the BP effects. Here the results indicated that on days when AARC-losses and stress severity were higher than the within-person average across days, negative affect also tended to be higher. The evidence for a negative association for WP AARC-gains was less clear, with the distribution of parameter estimates overlapping the ROPE and a smaller effect size relative to WP stress severity and WP AARC-losses. However, with over 80% of the distribution of credible values falling outside the ROPE, the evidence was in favor of a non-zero association, indicating that negative affect tended to be higher on days when AARC-gains was higher relative to the within-person average.

Of central interest given our research questions, was whether daily fluctuations in AARC-losses and AARC-gains moderated associations of daily stress with negative affect. More specifically, we expected that reactivity to stress (in the form of higher negative affect) would be greater on days when AARC-losses was higher than the within-person average, and weaker on days when AARC-gains was higher than the within-person average across days. Results of the two-way WP stress severity × WP AARC (gains and losses) interactions used to test these hypotheses are shown in Table 2. The results were strongly indicative of no two-way interactions. The distributions of credible values for both interaction terms fell almost entirely within the conventional ROPE (± 0.05) with 99 and 97% probability of a null effect for WP AARC-losses × stress interactions and WP AARC-gains × stress interactions, respectively. Even when relaxing the ROPE to ± 0.025, two-thirds or more of the credible estimates fell within the ROPE for each interaction term.

Finally, we tested the 3-way interaction of WP AARC-gains × WP AARC-losses × WP stress-severity. There was strong evidence in favor of the null (i.e., a zero-equivalent effect) when using the conventional ROPE_0_._05_ (P_null_ = 0.96); however, when applying the adjusted ROPE_0_._025_ there is evidence in favor of a negative non-zero effect with 81% of the falling outside the adjusted ROPE. Thus, we concluded that there is some evidence of a small, yet non-zero 3-way interaction trend in the data.

**FIGURE 2:**
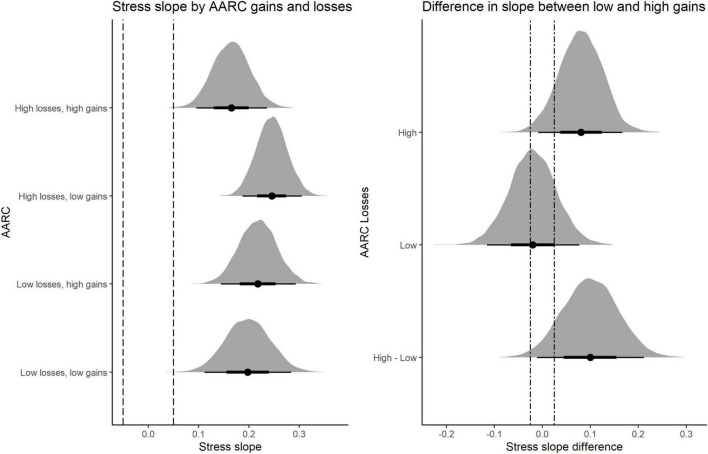
Left panel shows reactivity slopes (i.e., WP stressor severity) for combinations of lower (−1 *SD*) and higher (+ 1 *SD*) WP AARC-gains and WP AARC-losses. Right panel shows the difference between reactivity slopes for lower and higher WP AARC-losses, separately for lower and higher WP AARC-gains as well as higher minus lower AARC-gains. The latter value reflects the effect size of the 3-way interaction. The dashed lines represent the ROPE_0.05_ and the dot-dash line indicates the ROPE_0.025_.

To probe the nature of the 3-way interaction, the WP stress severity parameter (or *reactivity slope*) was estimated for individuals with different combinations of high (+ 1 *SD*) and low (−1 *SD*) WP AARC-gains and WP AARC-losses. Results of these analyses are shown in slope-on-a-ROPE, Figure 2, the left panel of which displays posterior distributions of the reactivity slopes (i.e., WP Stressor severity effects) for different combinations of WP AARC-gains and WP AARC-losses. The results indicate non-zero positive values for stress reactivity for each combination. We expected that the positive association of WP AARC-losses with stress reactivity would be weaker on days when AARC-gains was higher relative to the within-person average across days. This pattern was broadly evident in our data. On days when participants reported lower than usual AARC-losses, stress reactivity was moderate, irrespective of AARC-gains. However, on days when AARC-losses was relatively higher, those reporting low AARC-gains appeared more reactive to daily stressor severity. This pattern is confirmed in the right panel of Figure 2. It shows the difference in stress slope between low and high AARC-gains (i.e., low gains—high gains) separately by level of AARC-losses, as well as the difference between these differences (the value reflective of the effect size of the 3-way interaction).

### Vitality

**FIGURE 3:**
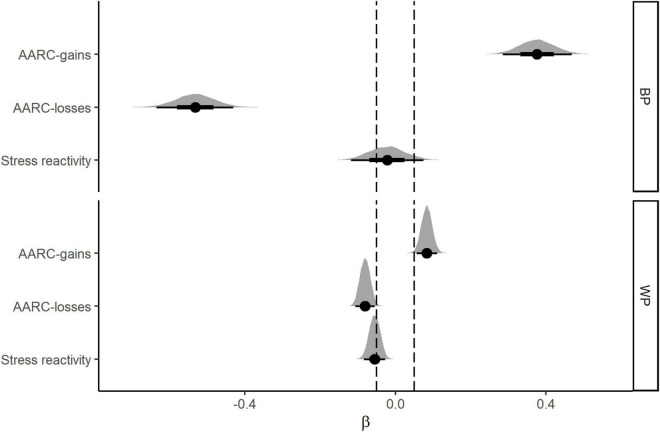
Between-person (BP, **top panel**) and Within-Person (WP, **bottom panel**) main effects for AARC and stress severity as predictors of vitality. The figure shows posterior distributions of coefficient estimates for the main effects and their degree of overlap with the region of practical equivalence (ROPE_0.05_).

Results from the Bayesian mixed-effects models used to examine associations of daily stress and AARC with vitality are shown in Table 3. The pattern of BP and WP main effects for vitality is illustrated in Figure 3. Results for the BP main effects revealed a negative coefficient for BP AARC-losses, and a positive coefficient for BP AARC-gains (Table 3). Both coefficients were non-zero (100% of each distribution of credible values fell outside the ROPE) indicating that participants who on average reported higher AARC-losses also reported lower vitality, whereas those who reported higher average AARC-gains reported higher vitality. For the BP stress severity effect, the evidence favored the null, with over 65% of the distribution falling within the ROPE.

**TABLE 3:** Results of Bayesian hierarchical mixed model predicting vitality.

	HDI_95%_				ROPE_0.05_			ROPE_0.025_		
Predictor	Est.	Error	Low	High	Below	Within	Above	Below	Within	Above
Intercept	0.07	0.07	−0.06	0.19						
Age	0.09	0.05	−0.00	0.19						
Female	−0.18	0.05	−0.27	−0.09						
Tertiary educated	0.03	0.04	−0.05	0.12						
Not in the labor force	−0.03	0.06	−0.15	0.1						
Physical functioning	0.04	0.05	−0.06	0.15						
Day in study	−0.07	0.06	−0.19	0.05						
Day in study^2^	0.06	0.06	−0.06	0.18						
Weekend	0.01	0.02	−0.02	0.04						
BP AARC-gains	0.38	0.05	0.29	0.47	0.00	0.00	1.00			
BP AARC-losses	−0.53	0.05	−0.63	−0.43	1.00	0.00	0.00			
BP stress severity	−0.02	0.05	−0.12	0.07	0.29	0.64	0.07			
WP AARC-gains	0.08	0.01	0.06	0.11	0.00	0.01	0.99			
WP AARC-losses	−0.08	0.01	−0.11	−0.05	0.99	0.01	0.00			
WP stress severity	−0.06	0.01	−0.08	−0.03	0.63	0.37	0.00			
WP AARC gains × WP AARC losses	0.00	0.01	−0.02	0.03						
WP AARC-gains × WP stress severity	−0.00	0.01	−0.03	0.02	0.00	1.00	0.00	0.04	0.94	0.02
WP AARC-losses × WP stress severity	−0.01	0.01	−0.03	0.02	0.00	1.00	0.00	0.11	0.89	0.00
WP gains × WP losses × WP stress severity	0.02	0.01	−0.00	0.04	0.00	1.00	0.00	0.00	0.29	0.71
Random intercept (*SD*)	0.48	0.01	0.46	0.50						

HDI, Highest Density Interval; Est, Estimate; SD, Standard Deviation; BP, Between-person; WP, Within-Person. Predictor and outcome variables were standardized prior to analysis.

Analyses of WP effects showed strong evidence for a non-zero association of WP AARC-losses with vitality, with vitality scores lower on days when AARC-losses was higher relative to the within-person average across days (P_meaningful_ = 99%). Similarly, WP AARC-gains was associated with higher daily vitality (P_meaningful_ = 99%). Consistent with the negative affect analysis reported above, WP effect sizes were substantially smaller than BP effect sizes. The evidence regarding the association between WP stress severity and vitality was equivocal. The HDI overlapped the ROPE, and the probability estimates did not clearly favor a meaningfully sized effect (63%) over a null effect (37%).

Our tests of 2-way interactions did not reveal evidence in favor of WP AARC-gains or WP AARC-losses moderating associations of daily stress severity with vitality (93 and 88% of the distributions fell within the ROPE for WP AARC-gains × WP stress severity and WP AARC-losses × stress severity, respectively). Finally, there was not compelling evidence in support of a 3-way interaction of WP AARC-gains × WP AARC-losses × WP stress severity. The coefficient HDI_95%_ fell completely within the conventional ROPE (± 0.05), and when applying a less stringent criterion (± 0.025) the HDI overlapped the ROPE, with close to 30% of the plausible estimates of the three-way interaction coefficient within the ROPE.

### Additional analyses

Recognizing that exposure to stressors might result in decreases in pleasant emotions as well as increases in unpleasant emotions [see (65)], we also fitted a model that included affect balance [positive affect—negative affect; see (48)] as the dependent variable. The results essentially mirrored those reported above for negative affect (see Supplementary material and Table 1).

Finally, it is possible that exposure to at least moderate levels of daily stress could in part reflect engagement in activity, which could in turn have implications for daily wellbeing. For example, a greater engagement with life is likely to be associated with greater exposure to interpersonal stressors (66) which could result in above average daily stress severity scores. We therefore conducted follow-up analyses including quadratic terms for BP and WP stress to examine possible non-linear associations. We were specifically interested in the possibility that daily negative affect and (lower) vitality would be associated with both relatively higher stress, as well as the absence of stress (implying a possible absence of meaningful engagement).

Focusing first on the analysis of negative affect, fit statistics did not reveal evidence for superior model fit resulting from the addition of WP quadratic stress and its interactions with the WP AARC variables (original model elpd = −1144.6, quadratic model elpd = −1146.7, eldp difference = −2.1, SE_difference_ = 3.7^1^).

Further examination of the model coefficients revealed some weak evidence suggesting that WP quadratic stress was non-zero (WP Stress linear estimate = 0.18, error = 0.03, P_meaningful_ = 100%; WP Stress quadratic estimate = 0.06, error = 0.03, P_meaningful_ = 66%), indicating a steeper incline in negative affect at higher levels of WP stress. However, evidence for non-zero effects among interaction terms including AARC variables and the WP stress quadratic was weak or favored the null (for ROPE_0_._025_, P_null_ all ≥ 38%).

**FIGURE 4:**
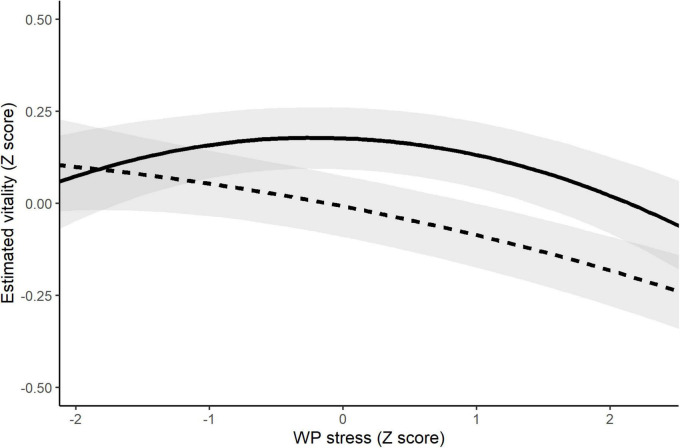
Interaction of quadratic WP Stress severity × WP AARC-losses in the prediction of vitality. The dashed line represents the association of WP stress with vitality at occasions when AARC-losses was higher (+ 1 *SD*) relative to the person-mean across days. The solid line represent the association of WP Stress with vitality on occasions when AARC-losses was lower (−1 *SD*) relative to the person-mean across days.

Results of the analysis that included vitality as the dependent variable (see Supplementary material and Table 2) also showed equivocal model fit statistics (original model elpd = −961.8, quadratic model elpd = −970.3, eldp difference = −8.5, SE_difference_ = 4.4). However, examination of the model estimates revealed stronger evidence of non-linear associations involving WP stress; specifically there was weak evidence supporting a quadratic WP Stress × WP AARC-losses interaction (P_meaningful_ = 74%). The nature of the interaction is illustrated in Figure 4 which shows associations of quadratic WP Stress with vitality at higher (+ 1 *SD*) and lower (−1 *SD*) levels of WP AARC-losses. As can be seen from Figure 4, when AARC-losses was relatively higher compared with the within-person average across days, the association between WP stress and vitality was close to linear, with greater stress relative to one’s average associated with lower vitality. In contrast, when AARC-losses was relatively lower compared with the within-person average across days, the association of WP Stress with vitality was curvilinear, with relatively higher vitality associated with average levels of daily stress, and less clear evidence for a decline in vitality associated with higher levels of daily stress.

## Discussion

The current study investigated whether midlife and older adults’ affective reactivity to daily stressors varied as a function of dynamic fluctuations in AARC. We expected that participants reporting higher AARC-gains and lower AARC-losses overall would also report lower negative affect and higher vitality (i.e., BP associations). We also predicted similar associations at the WP level and examined moderating effects; specifically, whether stress reactivity (in terms of higher negative affect and lower vitality in response to stressor exposure) would be buffered by higher WP AARC-gains, and whether daily associations of WP AARC-losses with stress reactivity would be less evident on days when AARC-gains was higher. Overall, our results provided support for some, but not all our hypotheses. We address the specific findings in turn in the sections that follow.

### Between- and within-person associations of awareness of age-related change with negative affect and vitality

Our analysis of between-person associations revealed that participants reporting overall higher AARC-gains and lower AARC-losses also reported lower negative affect and higher vitality. Similarly, at the within-person level, on days when AARC-gains was higher, and AARC-losses was lower, this corresponded with lower negative affect and higher vitality. These findings are broadly consistent with the growing body of empirical evidence linking subjective aging conceptualized within the AARC framework with wellbeing outcomes [e.g., (2, 4)]. With most of the existing research on AARC having been conducted with older samples from the United States and Europe, our findings speak to a similar relevance of these concepts to subjective aging and wellbeing in an Australian cultural context.

Our results pertaining to within-person associations also align with a growing body of recent empirical work indicating that subjective age may have state- as well as trait-like components, and that short-term fluctuations in views on aging, are temporally linked with short-term variation in other psychosocial variables. For example, Armenta et al. (68) reported substantial daily variations in subjective age bias and age-group identification in a sample of older workers. Moreover, daily fluctuations in the indices of subjective aging were associated with attributions of negative work-related events to age. Another daily diary study (69) reported that 23% of variability in felt age discrepancy (i.e., felt age—chronological age) was within-person; a level of within-person variation consistent with our findings for AARC-gains and AARC-losses. More recently, results of an ecological momentary assessment study indicated that subjective age can vary from moment-to-moment within a day, with similar degrees of variability observed among young-old and old-old adults (70). Studies have also shown that daily stressor exposure is related to daily felt age among older adults not exposed to higher levels of negative life events (71), and that higher daily AARC-losses is associated with higher daily negative affect (5). Higher-than-usual AARC-losses may align with more negative evaluations of one’s ability to handle current stressors (28), or may reflect reduced confidence in exercising self-regulatory agency through goal pursuit (31, 32). The extent to which daily stressors were attributed to aging was not measured in the current study. However, findings may indicate that stressors which threaten one’s self-awareness in ways that relate to subjective aging (e.g., having to limit or avoid activities as a result of age-related health problems) may pose particular risks for affective wellbeing in later life. Considering our findings in the context of the broader literature supports the idea that views on aging can vary across short time scales, perhaps in response to proximal reference points such as health-related events (1, 72).

Our study contributes to an emerging literature on short-term variability in subjective aging in several important ways. First, it is the only study that we are aware of two explicitly focus on the correspondence between daily fluctuations in AARC-gains and daily aspects of affective wellbeing. Recent efforts at designing health promotion interventions have drawn on evidence from the field of subjective aging (73), and we regard AARC-gains as an aspect of subjective age that has significant potential as a target for intervention. Recognizing age-related gains could have particular value in serving emotion-regulatory and motivational goals in the presence of objective and unalterable age-related losses (32). The coupling of AARC-gains with lower daily negative affect and higher vitality observed in the current study provides further evidence supporting a possible adaptive function of the awareness of positive aspects of aging.

Second, ours is the first study that we are aware of to examine linkages between short term fluctuations in AARC and vitality. Recent empirical (40) and theoretical work (74) has identified the centrality of energy as a resource for investment into goal-related activity across the lifespan, that might become subjectively more limited in older age. In their conceptual outline of AARC, Diehl and Wahl (21) postulate that AARC affects wellbeing outcomes *via* its influence on intervening self-regulatory processes. Recognizing the role of energy/vitality in shaping goal-directed behavior (40), our findings support the possibility that AARC-gains could help to sustain feelings of subjective vitality, whereas AARC-losses could lead to a more rapid diminishing of energy (as a side note, it is worth mentioning that the WP main effects reported here showed similar effect sizes for AARC-gains and AARC-losses in the prediction of vitality). In turn, such effects on energy could influence levels of engagement in adaptive self-regulatory behavior such as re-engagement with new goals in response to developmental losses [see (75)]. Reverse causal mechanisms are also plausible- for example, poor health on a given day could result in reduced energy levels that in turn increase the salience of age-related losses. Examining possible mediational links between AARC, subjective energy/vitality and self-regulatory behavior could represent a fruitful avenue for future studies.

### Moderating effects of awareness of age-related change-gains and awareness of age-related change-losses on stress reactivity

Contrary to our hypotheses, our results did not produce consistent evidence in support of WP AARC-gains or WP AARC-losses individually moderating associations of WP stressor severity with negative affect or vitality. However, when we included quadratic WP Stress in follow-up analyses to allow for possible non-linear associations, there was weak evidence for a WP Stress × AARC-losses interaction, which indicated that when AARC-losses was higher relative to the within-person average across days, the association of WP Stress with vitality was negative and essentially linear. In contrast, when AARC-losses was lower relative to the within-person average across days, the highest levels of vitality were evident at around average levels of stress severity, with comparatively lower vitality at both lower and higher levels of stress severity. This finding offers some preliminary evidence to suggests that when losses are more salient than usual, energy might be best preserved in the absence of activities with the potential to cause stress. In contrast, when older adults perceive their own aging in less negative terms than is typical for them, some degree of stress, perhaps reflecting the engagement in meaningful or challenging activities [see (66, 76)] may be associated with higher energy levels. As outlined above, viewing one’s own aging in a negative way could undermine effective self-regulation through inhibiting the mobilization of coping resources (29, 31). Our findings raise the possibility that viewing one’s own aging in less negative terms than is typical could facilitate older adults’ engaging in rewarding activities that promote vitality, and at the same time tolerate moderate levels of stress that may result from engagement in such activities (e.g., interpersonal tensions or disagreements). This interpretation is of course speculative- research that assesses activity engagement and use of coping processes in addition to AARC and stress exposure is needed to better understand the interplay of views on aging with stress and coping in shaping the development and maintenance of vitality in the daily lives of older adults. We also offer the further caveat here that the evidence in favor of this interaction effect was weak; clearly replication is needed.

When we applied an adjusted ROPE (see section “Results”), there was evidence for a trend in the data supporting a 3-way interaction of WP stress severity × WP AARC-gains × WP AARC-losses in the prediction of negative affect. The nature of the interaction suggested that on days when AARC-losses was higher than the within-person average across days, the presence of similarly higher AARC-gains may have resulted in less negative affective reactivity to stress exposure. This finding is broadly consistent with our recent cross-sectional work examining AARC-losses × AARC-gains interactions that points to the risks to wellbeing posed by AARC-losses being partially offset by the presence of AARC-gains (77). This buffering effect could emerge from AARC-gains promoting feelings of competence (78), positive self-image (21), or self-efficacy (79), that could assist in the mobilization of coping resources, or the use of putatively adaptive emotion-regulation strategies such as cognitive reappraisal [e.g., (80)]. The notion that the co-occurring experience of higher than typical AARC-gains with AARC-losses could help to protect against increases in negative affect is also broadly consistent with recent speculation about complexity in views on aging. Specifically, Kornadt et al. (1) suggest that a multifaceted representation of aging that is neither exclusively deficit- nor potential-oriented could better equip older adults to flexibly self-regulate in response to age-related challenges. We are, however, cautious to not overstate the significance of this finding; as was the case with the WP Stress × AARC-losses effect discussed above, evidence in favor of the interaction effect was weak. Further replication work is needed to establish the robustness of AARC-gains as a buffer of associations between AARC-losses and poorer wellbeing outcomes, as well as its potential role as a moderator of the effects of other well-established risk factors (e.g., poor physical health, social network losses) for poor wellbeing in older adulthood.

Overall, findings suggest that AARC varies over short time scales, and may be implicated in processes of affective reactivity to stress in later life. Recent research has shown that cognitive behavioral therapy techniques focused on recognizing and challenging aging stereotypes were successful in changing adults’ overall self-perceptions of aging (81). Accordingly, interventions that assist individuals to notice or retain positive perceptions of aging (along with related psychological resources e.g., competence, perceived control, self-efficacy), or avoid the salience of AARC-losses in the context of day-to-day life, could assist in preserving affective wellbeing in later life.

### Limitations and future directions

Our results should be considered alongside several limitations. First, the study design required participants to own and operate smartphones. Those confident in their smartphone use and willing to participate in this research may not be broadly representative of the general population of middle-aged and older adults. Future research should focus on whether the reported associations between AARC and stress reactivity are evident in more diverse samples such as those who are less confident with technology, or among the oldest-old (aged over 85 years) for whom AARC-losses may be increasingly salient, and AARC-gains may be more infrequent (82). Additionally, data collection occurred during the relatively early months (May-June 2020) of COVID-19 being declared a global pandemic by the World Health Organization. While Australia (and South Australia in particular) experienced overall less-extreme consequences in the first year of the pandemic (e.g., lower infection rates/fatalities) relative to other countries, participants’ daily lives were likely disrupted to some degree during data collection and this may have had implications for self-perceptions of aging (83).

While micro-longitudinal study designs provide high ecological validity, reduce recall bias and allow more nuanced understanding of relationships between AARC, stressors, and wellbeing than cross-sectional research, the nature of our data remains correlational, limiting the ability to determine causal relationships for the constructs of interest. As self-perceptions of aging and AARC may be modifiable through intervention [e.g., (73, 81)], relationships between AARC and wellbeing should be further investigated with experimental studies, or by modeling lagged or cross-correlational effects to provide evidence regarding the causal directionality of effects (84, 85). Finally, there is some controversy regarding the extent to which constructs related to subjective aging are conceptually distinct from more general dispositions toward viewing the world in a positive way [e.g., (86)]. Although we did not tap into the unique associations of AARC with affect and vitality in the present study by simultaneously controlling for relevant markers of psychological wellbeing (e.g., optimism, self-efficacy), future studies may benefit from such an approach.

## Conclusion

The present study contributes knowledge regarding individual differences and within-person fluctuations in AARC and wellbeing in middle- and older-adulthood. Our results showed that AARC-gains and AARC-losses were reliably associated with daily negative affect and vitality at both the between- and within-person levels. There was a trend to suggest that the vulnerability to negative affect arising from a combination of stressor exposure and AARC-losses could be offset by higher AARC-gains. Follow-up analyses also revealed that the daily coupling of stressor severity with lower vitality may be less evident on occasions when AARC-losses are lower than usual; however, both interaction effects were weak and require replication. Future research should examine associations between AARC and stress reactivity in more diverse samples, using experimental or time-lagged study designs, and consider potential processes through which AARC may have implications for stressor reactivity, including the roles of subjective energy and self-regulatory behavior.

## Data availability statement

The raw data supporting the conclusions of this article will be made available by the authors, without undue reservation.

## Ethics statement

The studies involving human participants were reviewed and approved by the Flinders University Human Research Ethics Committee. Written informed consent for participation was not required for this study in accordance with the national legislation and the institutional requirements.

## Author contributions

BW-H contributed to the study design, data analysis, and drafted an initial version of the manuscript. NW analyzed the data and contributed to writing the manuscript. TW contributed to the study design and analysis and drafting of the manuscript. All authors contributed to the article and approved the submitted version.

## Footnotes

1 We evaluated model fit *via* estimated log probability density (elpd) computed *via* leave one out cross validation. The elpd is an index of out-of-sample model fit with higher values reflecting better fit. The difference in fit between two models can be considered reliable when the elpd difference is more than twice the standard error of the difference [see (67)].
