# Supplementary material for: Awareness of age-related gains and losses as moderators of daily stress reactivity in middle- and older-adulthood
Source: Front Psychiatry. 2022 Aug 26;13:929657. doi: 10.3389/fpsyt.2022.929657 (PMC9458888; doi:10.3389/fpsyt.2022.929657)
Supplement: Supplementary file 1 [file Data_Sheet_1.docx]

**Supplementary Table 1.** *Results of Bayesian hierarchical mixed model predicting affect balance*

|  |  |  | HDI_95%_ | | ROPE_0.05_ | | | ROPE_0.025_ | | |
| --- | --- | --- | --- | --- | --- | --- | --- | --- | --- | --- |
| Predictor | Est. | Error | Low | High | Below | Within | Above | Below | Within | Above |
| Intercept | 0.02 | 0.07 | -0.12 | 0.16 |  |  |  |  |  |  |
| Age | 0.11 | 0.05 | 0.01 | 0.21 |  |  |  |  |  |  |
| Female | -0.08 | 0.05 | -0.18 | 0.02 |  |  |  |  |  |  |
| Tertiary Educated | 0.04 | 0.05 | -0.06 | 0.13 |  |  |  |  |  |  |
| Not in the Labour Force | 0.06 | 0.07 | -0.08 | 0.19 |  |  |  |  |  |  |
| Physical Functioning | -0.07 | 0.06 | -0.18 | 0.04 |  |  |  |  |  |  |
| Day in study | 0.08 | 0.06 | -0.03 | 0.19 |  |  |  |  |  |  |
| Day in study^2^ | -0.06 | 0.06 | -0.18 | 0.05 |  |  |  |  |  |  |
| Weekend | 0.03 | 0.01 | 0.00 | 0.06 |  |  |  |  |  |  |
| BP AARC-gains | 0.38 | 0.05 | 0.28 | 0.47 | .00 | .00 | 1.00 |  |  |  |
| BP AARC-losses | -0.42 | 0.06 | -0.53 | -0.31 | 1.00 | .00 | .00 |  |  |  |
| BP Stress severity | -0.19 | 0.05 | -0.30 | -0.09 | 1.00 | .00 | .00 |  |  |  |
| WP AARC-gains | 0.07 | 0.01 | 0.04 | 0.09 | .00 | .07 | .93 |  |  |  |
| WP AARC-losses | -0.10 | 0.01 | -0.13 | -0.07 | 1.00 | .00 | .00 |  |  |  |
| WP stress severity | -0.13 | 0.01 | -0.16 | -0.11 | 1.00 | .00 | .00 |  |  |  |
| WP AARC gains X WP AARC losses | 0.01 | 0.01 | -0.01 | 0.03 |  |  |  |  |  |  |
| WP AARC-gains X WP stress severity | 0.00 | 0.01 | -0.03 | 0.02 | .00 | 1.00 | .00 | .03 | .96 | .01 |
| WP AARC-losses X WP stress severity | -0.01 | 0.01 | -0.03 | 0.02 | .00 | 1.00 | .00 | .07 | .92 | .01 |
| WP gains X WP losses X WP stress severity | 0.03 | 0.01 | 0.01 | 0.05 | .00 | .98 | .02 | .00 | .02 | .98 |
|  |  |  |  |  |  |  |  |  |  |  |
| Random Intercept (*SD*) | 0.46 | 0.01 |  |  |  |  |  |  |  |  |

Note. HDI = Highest Density Interval. Est. = Estimate. *SD* = Standard Deviation. BP = Between-Person. WP = Within-Person. Predictor and outcome variables were standardised prior to analysis.

**Supplementary Table 2.** *Results of Bayesian hierarchical mixed model predicting vitality with inclusion of quadratic terms for BP Stress severity and WP Stress severity*

|  |  |  | HDI_95%_ | | ROPE_0.05_ | | | ROPE_0.025_ | | |
| --- | --- | --- | --- | --- | --- | --- | --- | --- | --- | --- |
| Predictor | Est. | Error | Low | High | Below | Within | Above | Below | Within | Above |
| Intercept | 0.06 | 0.06 | -0.06 | 0.19 |  |  |  |  |  |  |
| Age | 0.09 | 0.05 | -0.01 | 0.18 |  |  |  |  |  |  |
| Female | -0.18 | 0.05 | -0.27 | -0.09 |  |  |  |  |  |  |
| Tertiary Educated | 0.03 | 0.04 | -0.06 | 0.12 |  |  |  |  |  |  |
| Not in the Labour Force | -0.02 | 0.06 | -0.15 | 0.10 |  |  |  |  |  |  |
| Physical Functioning | 0.04 | 0.05 | -0.06 | 0.14 |  |  |  |  |  |  |
| Day in study | -0.08 | 0.06 | -0.20 | 0.04 |  |  |  |  |  |  |
| Day in study^2^ | 0.06 | 0.06 | -0.05 | 0.18 |  |  |  |  |  |  |
| Weekend | 0.01 | 0.02 | -0.02 | 0.04 |  |  |  |  |  |  |
| BP AARC-gains | 0.38 | 0.05 | 0.29 | 0.46 | .00 | .00 | 1.00 |  |  |  |
| BP AARC-losses | -0.53 | 0.05 | -0.64 | -0.43 | 1.00 | .00 | .00 |  |  |  |
| BP Stress severity | 0.01 | 0.07 | -0.13 | 0.15 | .22 | .52 | .26 |  |  |  |
| BP Stress severity^2^ | -0.01 | 0.07 | -0.14 | 0.13 | .26 | .53 | .21 |  |  |  |
| WP AARC-gains | 0.08 | 0.01 | 0.05 | 0.11 | .00 | .01 | .99 |  |  |  |
| WP AARC-losses | -0.08 | 0.01 | -0.11 | -0.05 | .99 | .01 | .00 |  |  |  |
| WP stress severity | -0.04 | 0.02 | -0.08 | -0.01 | .32 | .69 | .00 |  |  |  |
| WP stress severity^2^ | -0.06 | 0.02 | -0.10 | -0.01 | .60 | .40 | .00 |  |  |  |
| WP AARC gains X WP AARC losses | 0.00 | 0.01 | -0.03 | 0.03 |  |  |  |  |  |  |
| WP AARC-gains X WP stress severity | -0.01 | 0.01 | -0.04 | 0.02 | .00 | 1.00 | .00 | .13 | .86 | .01 |
| WP AARC-losses X WP stress severity | -0.03 | 0.02 | -0.06 | 0.00 | .10 | .90 | .00 | .59 | .41 | .00 |
| WP AARC-gains X WP stress severity^2^ | 0.02 | 0.02 | -0.01 | 0.06 | .00 | .95 | .06 | .00 | .53 | .47 |
| WP AARC-losses X WP stress severity^2^ | 0.03 | 0.01 | 0.01 | 0.06 | .00 | .92 | .08 | .00 | .26 | .74 |
| WP gains X WP losses X WP stress severity | 0.02 | 0.01 | 0.00 | 0.04 | .00 | 1.00 | .00 | .00 | .22 | .78 |
| WP gains X WP losses X WP stress severity^2^ | -0.01 | 0.02 | -0.04 | 0.02 | .00 | 1.00 | .00 | .34 | .55 | .11 |
|  |  |  |  |  |  |  |  |  |  |  |
| Random Intercept (*SD*) | 0.48 | 0.01 |  |  |  |  |  |  |  |  |

Note. HDI = Highest Density Interval. Est. = Estimate. *SD* = Standard Deviation. BP = Between-Person. WP = Within-Person. Predictor and outcome variables were standardised prior to analysis.
